# First person – Kim Landry-Truchon and Nicolas Houde

**DOI:** 10.1242/dmm.048199

**Published:** 2020-12-29

## Abstract

First Person is a series of interviews with the first authors of a selection of papers published in Disease Models & Mechanisms, helping early-career researchers promote themselves alongside their papers. Kim Landry-Truchon is first author on ‘Deletion of *Yy1* in mouse lung epithelium unveils molecular mechanisms governing pleuropulmonary blastoma pathogenesis’, published in DMM. Kim is a research assistant in the lab of Lucie Jeannotte at Centre de recherche du CHU de Québec-Université Laval, Québec, Canada, investigating organ development and the regulatory networks involved. Nicolas is a research assistant in the same lab, investigating the role of master transcription factors during mouse development.


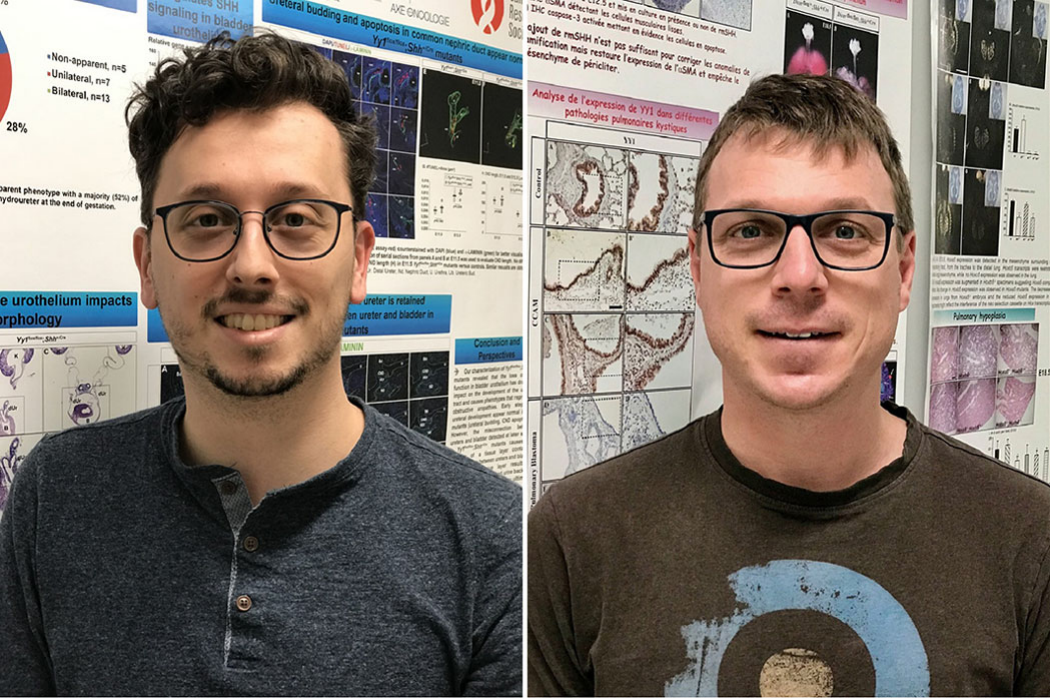


**Kim Landry-Truchon (left) and Nicolas Houde (right)**

**How would you explain the main findings of your paper to non-scientific family and friends?**

We are working on a very rare disease that is a lung cancer affecting young children, named pleuropulmonary blastoma (PPB). This disease starts during pregnancy and is due to genetic mutations in the *DICER1* gene, which encodes an enzyme essential for the production of microRNAs that are important for the expression of other genes. A predisposition mutation in *DICER1* is generally transmitted from a parent to the child. Subsequently, a random event happens in the second copy of the *DICER1* gene of the child, specifically in the lung. This leads to the formation of lung cysts that can eventually transform into a solid tumor, which can cause child death if not identified early and treated rapidly. One of the main challenges of PPB is its early detection, which is complicated by the fact that PPB symptoms are non-specific and often confounded with respiratory infections. PPB is also frequently mistaken for congenital pulmonary airway malformation (CPAM), another rare but non-tumorous cystic lung disease.

We have generated a mouse model that reproduces the early stage of PPB. We have shown that these mutant mice and human PPB samples share a common set of genes that are similarly deregulated, establishing a distinctive molecular signature. This molecular signature is specific to PPB and not observed in CPAM samples. Thus, these data represent the first step toward the understanding of the molecular mechanisms underlying PPB development.

“[…] these data represent the first step toward the understanding of the molecular mechanisms underlying PPB development.”

**What are the potential implications of these results for your field of research?**

Our long term goal is to contribute to improved diagnostic methods and better early management of the disease, and to identify molecular tools for the design of effective and specific therapies.

**What are the main advantages and drawbacks of the model system you have used as it relates to the disease you are investigating?**

We used a mouse model in which the *Yy1* gene was specifically abolished in the lung epithelium of mouse embryos. The main drawback of this mouse model is that mutant mice die at birth from respiratory distress, precluding any further analysis of tumor formation beyond birth. Moreover, the cystic lung phenotype is consequent to the loss of *Yy1* function in lung epithelium, while PPB is associated with mutations in the *DICER1* gene. Even though we have shown that *Yy1* expression is affected by the abnormal microRNA profile consequent to *DICER1* mutations, the *Yy1* mouse line does not reproduce the genetic status of the patients affected with PPB. On the other hand, the main advantage of our mouse model is its 100% penetrance. The identification of the molecular signature was facilitated by the analysis of mouse specimens. It would have been more difficult to only study human material due to the scarcity and heterogeneity of PPB samples.

**Describe what you think is the most significant challenge impacting your research at this time and how will this be addressed over the next 10 years?**

The most significant challenge will remain the access to biological material from PPB patients. The design of a mouse model able to closely recapitulate PPB through its characteristic tumor pathological changes is therefore necessary.

**What's next for you?**

We plan to pursue our study of our current *Yy1* mutant mouse line and to produce a more accurate and precise PPB mouse model. Using a conditional gene targeting strategy combined with different Cre recombinase mouse lines, this new mouse model should allow the thorough characterization of PPB.

**Figure:**
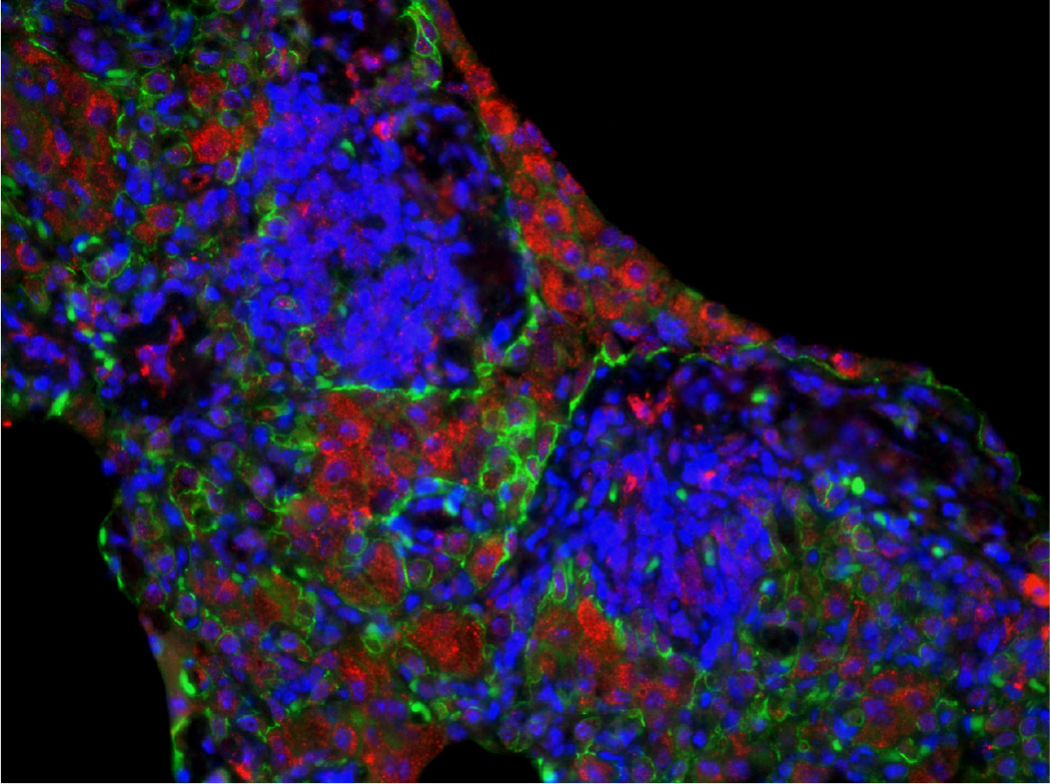
**Epithelial lung cysts from a rare surviving *Yy1*^flox/flox^;*Tg*^+/Nkx2.1Cre^ specimen.** FGF9 (red) and E-cadherin (green) proteins are identified and illustrate profound epithelial disorganization in the cystic wall of mutant mice. Cell nuclei are counterstained with DAPI.
